# Acceptance, needs and demands of e-mental health interventions in adolescent elite athletes

**DOI:** 10.1007/s44192-026-00474-9

**Published:** 2026-06-11

**Authors:** Sheila Geiger, Sara Viehweger, Vanessa Thuy Mi Nguyen, Jochen Seitz, Gertraud Gradl-Dietsch, Thomas Muehlbauer, Georgios Paslakis, Eva-Maria Skoda, Martin Teufel, Alexander Bäuerle, Anna Julia Esser-Seraphin

**Affiliations:** 1 Clinic for Psychosomatic Medicine and Psychotherapy, LVR-University Hospital Essen, University of Duisburg-Essen, Virchowstr. 174, 45147 Essen, Germany; 2 Center for Translational Neuro- and Behavioral Sciences (C-TNBS), University of Duisburg-Essen, 45147 Essen, Germany; 3 Department of Child and Adolescent Psychiatry, Psychosomatics and Psychotherapy, LVR-University Hospital Essen, University of Duisburg-Essen, Essen, Germany; 4 Division of Movement and Training Sciences, Biomechanics of Sport, University of Duisburg-Essen, Essen, Germany; 5 Medical Faculty, University Clinic for Psychosomatic Medicine and Psychotherapy, Campus East-Westphalia, Ruhr‐University Bochum, Lübbecke, Germany

**Keywords:** eHealth, Digital health, UTAUT, Sports, Mental health, Youth

## Abstract

**Background:**

Elite sports place high psychological and physical demands on adolescent elite athletes, increasing the risk for mental health problems. E-mental health interventions may offer flexible and low-threshold support. This study investigated adolescent elite athletes’ acceptance of e-mental health interventions, identified predictors of acceptance, and explored their specific needs and demands.

**Methods:**

Data from *N* = 217 adolescent elite athletes (age range: 14–17 years) participating in a web-based survey (March 2022–March 2023) were analyzed. Frequencies for preferred program features and delivery modes were calculated. Acceptance and its predictors were examined using an extended Unified Theory of Acceptance and Use of Technology (UTAUT) model. Group comparisons (*t*-tests, ANOVAs) and hierarchical regression analyses were conducted.

**Results:**

Overall acceptance of e-mental health interventions was moderate. Female athletes and those in individual sports indicated higher acceptance than male and team-sport athletes. Gender (*β* = 0.097, *p* = .017), the UTAUT predictors performance expectancy (*β* = 0.458, *p* < .001), effort expectancy (*β* = 0.309, *p* < .001), and social influence (*β* = 0.152, *p* = .015) significantly explained variance in acceptance in adolescent elite athletes, whereas sport type, depression-, anxiety-, and distress symptoms, internet anxiety, digital confidence, and -overload were not significant predictors. Preferred interventions were customizable, delivered via smartphone app, and completed in 10–20-minutes sessions.

**Conclusions:**

Findings highlight the need for user-centered, flexible e-mental health interventions tailored to adolescent elite athletes. Targeting key acceptance drivers and preferred design features may support sustained engagement and inform indicated prevention strategies in this population.

## Introduction

In addition to achieving peak physical performance, young elite athletes have to deal with a number of other challenges. They often experience significant impairments in psychological health [1, 2]. A substantial part of their time is dedicated to rigorous training in preparation for competitive events [3]. They must perform at a high level, whether in the context of national or international competition, or in pursuit of peak performance [4–7].

Athletes’ careers usually begin at a young age and reach their peak, on average, in their 20s [8]. Many adolescent elite athletes strive for success in their sport discipline [9], often under pressure by their social environment [9]. The continuous exposition to risks such as overtraining or injury can hinder their pursuit of success and contribute to the perception of failure [10–13]. In addition to these sport-specific stressors, they are exposed to the normative challenges of personal development, including the processes of puberty and the burdens imposed by social pressures from both their studies and personal lives [14]. These combined demands can contribute to the development of psychological symptoms and disorders like anxiety or depression [15–17]. Adolescence constitutes a crucial period during which many mental disorders first manifest [18–20]. Although mental disorders frequently originate in early life, they are often not recognized until several years later [19]. According to a national study of mental health and wellbeing, nearly 40.00% of adolescent participants aged 16–24 years were already experiencing a mental disorder that had persisted for at least one year [21]. According to another study, elite athlete 12–15 year old female students showed higher anxiety and depression symptoms compared to female non-athletes at the same age [22]. Thus, adolescent athletes may constitute a particularly vulnerable group, given their developmental stage and the highly performance-oriented environment in which they operate, highlighting the importance of ensuring appropriate mental health support for this young population [23].

Despite this vulnerability, only a small proportion of adolescent elite athletes experiencing psychological or physical difficulties actively seek professional support. Barriers include fear of stigmatization and the concern that acknowledging such challenges may be perceived as a sign of weakness within their sporting context [24]. Limited mental health literacy [25] and restricted access to professional psychological services further impede help-seeking [24].

E-mental health interventions, or online-based psychological support, offer a promising alternative. E-mental health interventions provide educational resources, preventive information, and evidence-based support that can empower users [26, 27]. They may complement or substitute in-person therapy, offering benefits such as reduced waiting times [28]. Moreover, an e-mental health intervention enables cost-effective delivery [4], and flexible integration into demanding training schedules [29, 30]. For adolescent elite athletes, e-mental health interventions are particularly relevant, as many adolescents are likely to prefer text-based communication over verbal modalities when seeking help [31], potentially helping to overcome reservations about initiating psychological support [32].

Prior to developing and implementing e-mental health interventions, the assessment of their acceptance within the target population is required to inform design decisions and promote subsequent uptake [33]. Previous studies have examined adolescents non-athletes’ and adult elite athletes’ acceptance of e-mental health interventions [34–36], while research on acceptance among adolescent elite athletes remains limited [37]. In the case of adult elite athletes, the level of acceptance for e-mental health interventions is high [35]. Furthermore, the level of acceptance was found to vary depending on factors like gender, type of sport, and previous experiences with e-mental health interventions [35, 38]. The Unified Theory of Acceptance and Use of Technology (UTAUT) has been identified as a useful model for assessing the acceptance of eHealth interventions across multiple studies and is applied in the present study to better understand acceptance within the target group [33, 37]. The UTAUT assesses acceptance, defined as the intention to use e-mental health interventions, and its predictors [39]. The predictor Performance expectancy (PE) denotes the degree to which an individual perceives that using a technology will yield benefits [40]. Effort Expectancy (EE) refers to the perceived ease of using the technology, with higher acceptance when it is considered simple to use [40]. Another important determinant is social influence (SI), which refers to the extent to which an individual’s social environment, such as trainers, friends, and parents, supports and encourages the use of the intervention [35, 40, 41]. The adoption of technology is known to be influenced by positive feedback or recommendations from trusted individuals [40]. Among various studies, the UTAUT predictors have been shown to be significant and highly influential factors for acceptance [35, 42–44].

Previous research highlights the importance of assessing the expectations and preferences of potential users during the process of developing e-mental health interventions [45, 46]. In order to prevent non-adherence, e-mental health interventions should be developed according to the specific needs and demands of adolescent elite athletes [47].

In line with this premise, the present study focuses on the following objectives. The primary objective of this study is to explore the level of acceptance of e-mental health interventions among adolescent elite athletes. The second objective is to ascertain whether acceptance levels vary based on sociodemographic or sport-related factors. The third objective is to assess the factors that influence acceptance among adolescent elite athletes. The fourth objective is to identify adolescent elite athletes’ needs and demands regarding features, programmes and availability of an e-mental health intervention.

## Methods

### Study design and participants

The research was conducted as a web-based cross-sectional study among adolescent elite athletes. Two-hundred forty-eight participants were reached via regional and national sport clubs and associations, social media and coaches. The survey was designed using Unipark software (Tivian XI GmbH) and approved by the Ethics Committee of the Medical Faculty of the University of Duisburg-Essen (19-8947-BO). Electronic informed consent was obtained from all participants before editing the survey and the participation was anonymous and voluntary, with no financial or material incentives offered. Information materials (e.g., brochures) were provided for legal representatives. The criteria for adolescent (age between 14 and 17 years) elite athletes were as follows: alignment of life to sports, striving for excellence, and participation in professional or Olympic competitions [5–7]. Out of the *N* = 248 participants who answered the survey, *N* = 25 were younger than 14 years or older than 17 years (11.52%), and *N* = 6 did not meet the criteria of elite athletes (2.76%). The final sample size for data analysis comprised *N* = 217 adolescent elite athletes.

### Measures

#### Sociodemographic and sport-related data

The questions about the sociodemographic data included gender, chronological age, and living situation (for example dependent living situation or independent living situation). Sport related questions covered the sport discipline, years in elite sports, duration of a single training session, number of training sessions per week and days at home per month.

#### Psychometric data

The eight-item Patient Health Questionnaire depression scale (PHQ-8) is a valid measure to evaluate depression symptoms [48]. Depression symptoms were measured by the PHQ-8, which is an eight-item self-report instrument, rated on a scale from 0 (not at all) to 3 (almost every day). The PHQ-8 is a widely used and validated instrument for assessing depressive symptoms in both clinical and non-clinical populations [48]. The German versions of the PHQ instruments have been validated in population-based samples and have demonstrated good reliability (Cronbach’s *α* = 0.85) and construct validity in previous studies [35, 49]. The common cut-off score of 10 points was used to determine the proportion in the conspicuous value range [48]. Next, the Generalized Anxiety Disorder seven scale (GAD-7) was used to evaluate symptoms of generalized anxiety disorder [50]. The GAD-7 is a validated instrument with good reliability (Cronbach’s *α* = 0.79–0.91) and validity in previous studies [50, 51], including a German sample [52]. The items can be rated from 0 (not at all) to 3 (almost every day). It can be categorized in minimal (0–4), mild (5–9), moderate (10–14), and severe (15–21) levels of anxiety. The Distress Thermometer (DT) measures psychological distress in the past week on a visual scale from 0 (no distress) to 10 (extreme distress). This version is considered a validated instrument for screening distress with acceptable test-retest reliability (*r* = 0.80, *p* = 0.00) [53], discriminating clinically significant distress and its correlations with established measures of anxiety and depression [54, 55]. A cut-off score of 4 is approved to show relevant distress [55].

#### E-mental health-related data

To assess the participants’ experience with e-mental health interventions, self-developed items asked whether they were aware of such interventions and whether they had previously used them. Furthermore, information and communication technology-related questions about confidence in using digital media, internet anxiety and digital overload were asked, which have proven to be effective in previous studies [35, 56–58]. Their digital confidence was assessed using three items (use of digital media, online platforms, and digital devices) on a five-point Likert scale (1 = not confident at all, 5 = very confident) with good internal consistency (Cronbach’s *α* = 0.87). To assess internet anxiety, participants responded using a five-point Likert scale (concerns about using the internet), ranging from 1 (“does not apply to me”) to 5 (“does apply to me”). The internal consistency of this scale was found to be good (Cronbach’s *α* = 0.79). Furthermore, we utilised self-developed items to measure digital overload, which showed good internal consistency as well (Cronbach’s *α* = 0.79). Responses were collected on a five-point Likert scale, with questions such as “I feel burdened by the constant accessibility via cell phone or mail”, rated from 1 (“does not apply to me”) to 5 (“does apply to me”).

### Acceptance and UTAUT predictors

Applying the modified questionnaire based on the UTAUT model, acceptance towards e-mental health interventions was determined [35, 43]. The UTAUT-based questionnaire used in this study has been applied in previous research on e-mental health acceptance and has been validated in the context of technology acceptance, including e-mental health interventions [35, 39]. The internal consistency (Cronbach’s *α* = 0.90) of acceptance was found to be high in a previous German study [35]. The adapted UTAUT questionnaire includes 14 items rated on a five-point Likert scale, from 0 (strongly disagree) to 4 (strongly agree). Two UTAUT predictors, Effort Expectancy (EE), for instance and Social Influence (SI) were measured with three items each, showing adequate reliability in previous studies [59]. A further predictor, Performance Expectancy (PE) was measured with four items, showing excellent internal consistency (Cronbach’s *α* = 0.91) in prior research [35]. The study’s dependent variable, acceptance, was operationalized as behavioural intention (BI), assessed through four items [35].

### Needs and demands

Adolescent elite athletes indicated their intended frequency of use of the e-mental health intervention (1 = every day, 2 = once a week, 3 = every two weeks, 4 = once a month, 5 = only on demand). They also selected the type of e-mental health intervention that best matched their preferences, such as a short and intensive e-mental health intervention with multiple exercises per day over approximately four weeks, or a flexible, individually tailored e-mental health intervention providing more support at the beginning that gradually decreases over time. Adolescent elite athletes reported their preferred device for accessing the e-mental health intervention (e.g., computer, tablet, or smartphone), the desired duration of each session (e.g., 1–10 min, 10–20 min, 20–30 min, > 30 min), and the format of the content (e.g., interactive app or downloadable material). These items were based on validated instruments for assessing user needs and technology acceptance in e-mental health intervention research [44, 60–62]. In addition, based on previous research and in collaboration with experts in psychosomatic medicine, sports psychology, and digital health, a set of topics addressing mental health challenges and coping strategies in elite sports was compiled. These topics were rated by the adolescent elite athletes for inclusion in the e-mental health intervention [60, 63, 64]. The response format was a five-point Likert scale ranging from 0 (not relevant) to 4 (very relevant).

### Statistical analysis

The statistical analysis was conducted using IBM`s SPSS Statistics version 26 (New York, NY, USA, 2019) and RStudio version 4.0.2 (Boston, MA, USA, 2020). First, internal consistencies of the various psychometric questionnaires were assessed, and descriptive statistics were generated. Based on prior studies, acceptance was categorized into three levels: low (1.00-2.34), moderate (2.35–3.67), and high (3.68-5.00). Sum scores were calculated for the GAD-7 and PHQ-8 scales. To analyse mean acceptance differences across female and male adolescent elite athletes based on sociodemographic, sports, and e-mental health intervention-related factors, *t*-tests and ANOVAs were used; ANOVAs were applied to variables with multiple categories. The significance level was set at *α* = 0.05 (two-sided). *Post-hoc* tests were performed after mean comparisons, with Bonferroni correction applied to adjust *α*. Given the sample size (*N* = 217), normal distribution of variables was assumed based on the central limit theorem [65], allowing the use of parametric tests. Multiple hierarchical regression was conducted to assess the acceptance model, incorporating variables: (1) sociodemographic data, (2) mental health variables, (3) eHealth-related factors, and (4) UTAUT predictors. The first three steps were conducted to control for variables which also influence acceptance for e-mental health interventions. Extending the original UTAUT model by incorporating such factors has been demonstrated to improve its explanatory power compared to models including only core acceptance predictors [56]. In the final step, the UTAUT predictors were included as theory-based determinants of acceptance. This hierarchical approach enables the assessment of the incremental validity of the UTAUT model and followed approaches in previous studies to allow comparability of results [35, 42, 66]. Multicollinearity among predictors was evaluated, indicating no evidence of problematic multicollinearity. The distributional assumption of the residuals was evaluated, which showed no apparent deviations from normality, thus supporting the assumption of normally distributed residuals. The frequencies of the athletes’ statements on preferred functions, programs, and formats of the e-mental health intervention were calculated.

## Results

### Sample characteristics

Table 1 shows the demographic characteristics of the sample. In total, *N* = 217 participants were *M =* 15.74 years (*SD* = 1.05; 58.00% female, 41.00% male and 1.00% divers) old. The age distribution showed that 17.97% of the adolescent elite athletes were 14 years old, 29.95% were 15 years old, 28.57% were 16, and 23.50% were 17 years old. Eighty-one athletes participated in individual sports, while *n* = 93 were involved in team sports. On average, athletes had been involved in elite sports for *M* = 5.02 years (*SD* = 7.76), and their average number of training sessions was 5.73 times (*SD* = 2.58) per week. The average duration of a single training session amounted to 107.26 min (*SD* = 51.06) and the average days at home during the last month were 26.31 (*SD* = 19.96). The majority of athletes (94.01%) have a dependent living situation (with parents etc.).

**Table 1:** Demographic characteristics of the sample (*N* = 217)

Variable	*n*	%
*Types of sports*		
Ball sports	85	39.17
Combat sports	9	4.15
Strengths sports	2	0.92
Track and field	42	19.35
Equestrian sports	4	1.84
Gymnastics	10	4.61
Dance sports	5	2.30
Winter sports	3	1.38
Other sports	57	26.27
*Living situation*		
Dependent	204	94.01
Independent	1	0.46
Assisted living	3	1.38
Sports center	5	2.30
Other	4	1.84

*n*: sample size

Table 2 visualizes the prevalence of generalized anxiety symptoms, depression symptoms and psychological distress stratified by gender. Mild generalized anxiety symptoms were indicated by 25.81% of the adolescent elite athletes, moderate by 9.68%, and severe by 2.30%. The results of the questionnaire clarified that 21.66% of the athletes experienced symptoms of major depression. Using the cut-off score of four, 90.32% displayed relevant psychological distress.

**Table 2:** Prevalence of generalized anxiety symptoms, depression symptoms, and psychological distress stratified by gender

Questionnaire		Gender		
	Total	Female	Male	Divers
	*N* (%)	*n* (%)	*n* (%)	*n* (%)
*GAD-7*				
< 5	135 (62.22)	59 (46.83)	74 (84.09)	2 (66.67)
≥ 5	56 (25.81)	44 (34.92)	11 (12.50)	1 (33.33)
≥ 10	21 (9.68)	18 (14.29)	3 (3.41)	0
≥ 15	5 (2.30)	5 (3.97)	0 (0.00)	0
*PHQ-8*				
< 10	170 (78.34)	87 (69.05)	82 (93.18)	1 (33.33)
≥ 10	47 (21.66)	39 (30.95)	6 (6.82)	2 (66.67)
*DT*				
< 4	21 (9.68)	14 (11.11)	7 (7.95)	0
≥ 4	196 (90.32)	112 (88.89)	81 (92.04)	3 (100.0)
Total	*N* = 217	*n* = 126	*n* = 88	*n* = 3

*n*: sample size; GAD-7: Generalized Anxiety Disorder seven scale [50]; PHQ-8: Patient Health Questionnaire depression scale [48]; DT: Distress Thermometer [54]

### Research objective 1 and 2: acceptance of e-mental health Interventions

The general acceptance of e-mental health interventions was moderate to high (*M* = 3.30, *SD* = 1.17). In the following, the results of *N* = 217 adolescent elite athletes are classified by their level of acceptance: *n* = 47 (21.66%) athletes showed low acceptance, *n* = 57 (26.27%) athletes showed moderate acceptance, and *n* = 113 (52.07%) showed high acceptance. Table 3 displays the differences in acceptance by sociodemographic and sport specific data. Female athletes indicated significantly higher acceptance than male adolescent elite athletes. The median acceptance of adolescents, who identified outside the binary gender categories, was high (*Mdn* = 3.75, *range* = 2.00, 4.50). Also, the acceptance of the adolescent elite athletes participating in individual sports was higher compared to the adolescent elite athletes in team sports (see Table 3*).*

**Table 3:** Differences in acceptance (UTAUT behavioural intention scale) by sociodemographic and sport-specific data (*N* = 217)

Variable	*n*	%	M (SD)	Test	*p*-value	Effect size
*Sex*				*t*_212_= − 2.83	0.005*	Cohen‘s *d* = − 0.39
Female	126	58.06	3.50 (1.06)			
Male	88	40.55	3.03 (1.26)			
*Sports*				*F*_2,214_ = 4.27	0.015*	*η²* = 0.04
Individual Sport	81	37.33	3.58 (1.09)			
Team sport	93	42.86	3.08 (1.16)			
Individual and team sports	43	19.82	3.26 (1.22)			
*Experience with e-mental health*				*F*_2,214_ = 1.56	0.213	*η²* = 0.01
Received online support offers	6	2.76	3.79 (0.83)			
Knowledge about online support	35	16.13	3.54 (1.01)			
No knowledge about online support	176	81.11	3.24 (1.20)			

Group comparison (variable = sex) include male and female participants (*n* = 214); *: *p* < .05. *M*: mean; *SD*: standard deviation; *t*: *t* value; *F*: *F* value; UTAUT: Unified Theory of Acceptance and Use of Technology [43]. Effect sizes are reported as Cohen’s *d* for t-tests and eta squared (*η*²) for ANOVA

### Research objective 3: predictors of acceptance

Table 4 shows the results of the hierarchical regression model of acceptance. The multiple hierarchical regression analysis revealed that sociodemographic and sport-specific predictors explained 10.69% of the variance in acceptance (*R*^*2*^ = 0.107, *F*_4.209_ = 6.26, *p* < .001). The sociodemographic predictor gender (*β* = 0.097, *p* = .017) predicted acceptance significantly. The inclusion of mental health variables significantly increased explained variance (Δ*R*² = 0.024, *F*_3.203_ = 9.36, *p* < .001). Neither depression symptoms (*β* = − 0.006, *p* = 0.355), nor generalized anxiety (*β* = 0.056, *p* = .393) and distress (*β* = − 0.054, *p* = .162) predicted acceptance. Information and communication technology-related variables were added in the third part, which explained additional 3.59% of the total variance (Δ*R*² = 0.036, *F*_*3.203*_ = 9.36 *p* < 0.001). Digital overload was no significant predictor of acceptance (*β* = 0.039, *p* = .439), same as internet anxiety (*β* = 0.031, *p* = .502) and digital confidence (*β* = 0.016, *p* = 676). In the last step UTAUT predictors were included, which elucidated 57.69% (Δ*R²* = 0.577, *F*_3.200_ = 150.22, *p* < 0.001) of the variance. The UTAUT predictors PE (*β* = 0.458, *p* < .001), EE (*β* = 0.309, *p* < .001) and SI (*β* = 0.152, *p* = 0.015) were included and revealed themselves as significant predictors of acceptance. The total explained variance was therefore 73.80%.

**Table 4:** Hierarchical regression model for the prediction of acceptance (*N* = 214)

Predictor				β	B	t	*R*²	ΔR²	*p*	ANOVA
*Step 1: Sociodemographic and sport-specific predictors*							0.107			< .001^b^
Age				−0.043	-0.048	−1.076			0.238	
Gender				0.097	0.23	2.400			0.017*	
Sport type in reference to individual sport										
Teamsport				−0.057	−0.136	−1.381			0.169	
Both				−0.023	−0.066	−0.558			0.577	
*Step 2: Mental health variables*							0.131	0.024		< .0.001^c^
PHQ-8				−0.060	−0.015	−0.928			0.355	
GAD-7				0.056	0.016	0.856			0.393	
DT				−0.054	−0.027	−1.404			0.162	
*Step 3: Information and Communication Technology- related variables*							0.167	0.036		< .001^d^
Digital confidence				0.016	0.024	0.418			0.676	
Internet anxiety				0.031	0.042	0.672			0.502	
Digital Overload				0.039	0.046	0.776			0.439	
*Step 4: UTAUT predictors*							0.744	0.577		< .001^e^
PE				0.458	0.477	7.478			< 0.001	
EE				0.309	0.367	5.577			< 0.001	
SI				0.152	0.174	2.516			0.013	

In Step 2, 3 and 4, only the newly included variables are presented. *β*: standardized coefficient beta; B: unstandardized coefficient beta; *t*: *t*-value; *p: p*-value; *R*²: determination coefficient; Δ*R*²: Changes in *R*². GAD-7: Generalized Anxiety Disorder seven scale [50]; PHQ-8: Patient Health Questionnaire depression scale [48]; DT: Distress Thermometer [53]; UTAUT: Unified Theory of Acceptance and Use of Technology [43]; PE: Performance Expectancy; EE: Effort Expectancy; SI: Social Influence. Modelfit: *R²* = 0.744, adj. R² = 0.727, *F*_*13,200*_ = 44.70, *p* < .001.

### Research objective 4: adolescent elite athletes’ needs and demands regarding e-mental health intervention characteristics

Table 5 presents the preferred characteristics of e-mental health interventions stratified by sport type. Most adolescent elite athletes were interested in using e-mental health interventions on demand (41.47%), while 36.41% of the athletes intended to use the e-mental health intervention every week and 17.05% of athletes reported to use it daily. A short, individually customizable e-mental health intervention that offers support on several days per week and is gradually reduced was preferred by 51.15% of the athletes. However, 19.82% of the athletes would rather receive a task every day or every second day over two to three months. A long-term e-mental health intervention over six to twelve months (15.67%) and a short intense e-mental health intervention for a week (11.98%) received less favourability. A single session that requires between 10 and 20 min was chosen as the preferred duration by most of the adolescent elite athletes (47.00%).

**Table 5:** Reports from athletes on their needs and requirements regarding the preferred features and accessibility of e-mental health interventions (*N* = 217)

	*N* total = 217 *n* (*%*)	Individual sports *n* = 81 *n* (*%*)	Team sports *n* = 93 *n* (*%*)	Both sport types *n* = 43 *n* (*%*)
*Availability*				
Smartphone	207 (95.39)	78 (96.30)	89 (95.70)	40 (93.02)
Tablet/I-Pad	177 (81.57)	64 (79.01)	77 (82.80)	36 (83.72)
Computer	166 (75.50)	57 (70.37)	70 (75.27)	39 (90.70)
*Format*				
App	202 (93.09)	77 (95.06)	85 (91.39)	40 (93.02)
Website	99 (45.62)	33 (40.74)	44 (47.31)	22 (51.16)
Audio or video material	117 (53.92)	43 (53.07)	47 (50.54)	27 (62.79)
Interactive training	113 (52.07)	45 (55.56)	41 (44.09)	27 (62.79)
Downloadable materials	83 (38.25)	35 (43.21)	31 (33.34)	17 (39.53)
*Intended frequency of use*				
Hourly	1 (0.46)	0 (0)	1 (1.08)	0 (0.00)
Daily	37 (17.05)	22 (27.16)	12 (12.90)	3 (6.98)
Once a week	79 (36.41)	35 (43.21)	30 (32.26)	14 (32.56)
Once a month	10 (4.61)	3 (3.70)	5 (5.38)	2 (4.65)
Only on demand	90 (41.47)	21 (5.92)	45 (48.39)	24 (55.81)
*Session duration*				
1–10 min	37 (17.05)	13 (16.05)	19 (20.43)	5 (11.63)
10–20 min	102 (47.00)	44 (54.32)	39 (41.94)	19 (44.19)
20–30 min	64 (29.49)	21 (25.93)	27 (29.03)	16 (37.21)
> 30 min	6 (2.76)	1 (1.23)	2 (2.15)	3 (6.98)
Other	8 (3.69)	2 (2.47)	6 (6.45)	0 (0.0)
*Frequency of new content*				
Daily	1 (0.46)	0 (0)	1 (1.08)	0 (0)
Once a week	37 (17.05)	22 (27.16)	12 (12.90)	3 (6.98)
Every other week	79 (36.41)	35 (43.21)	30 (32.26)	14 (32.56)
Once a month	10 (4.61)	3 (3.70)	5 (5.38)	2 (4.65)
All content should be available from beginning	90 (41.47)	21 (25.93)	45 (48.39)	24 (55.81)

**Fig. 1:**
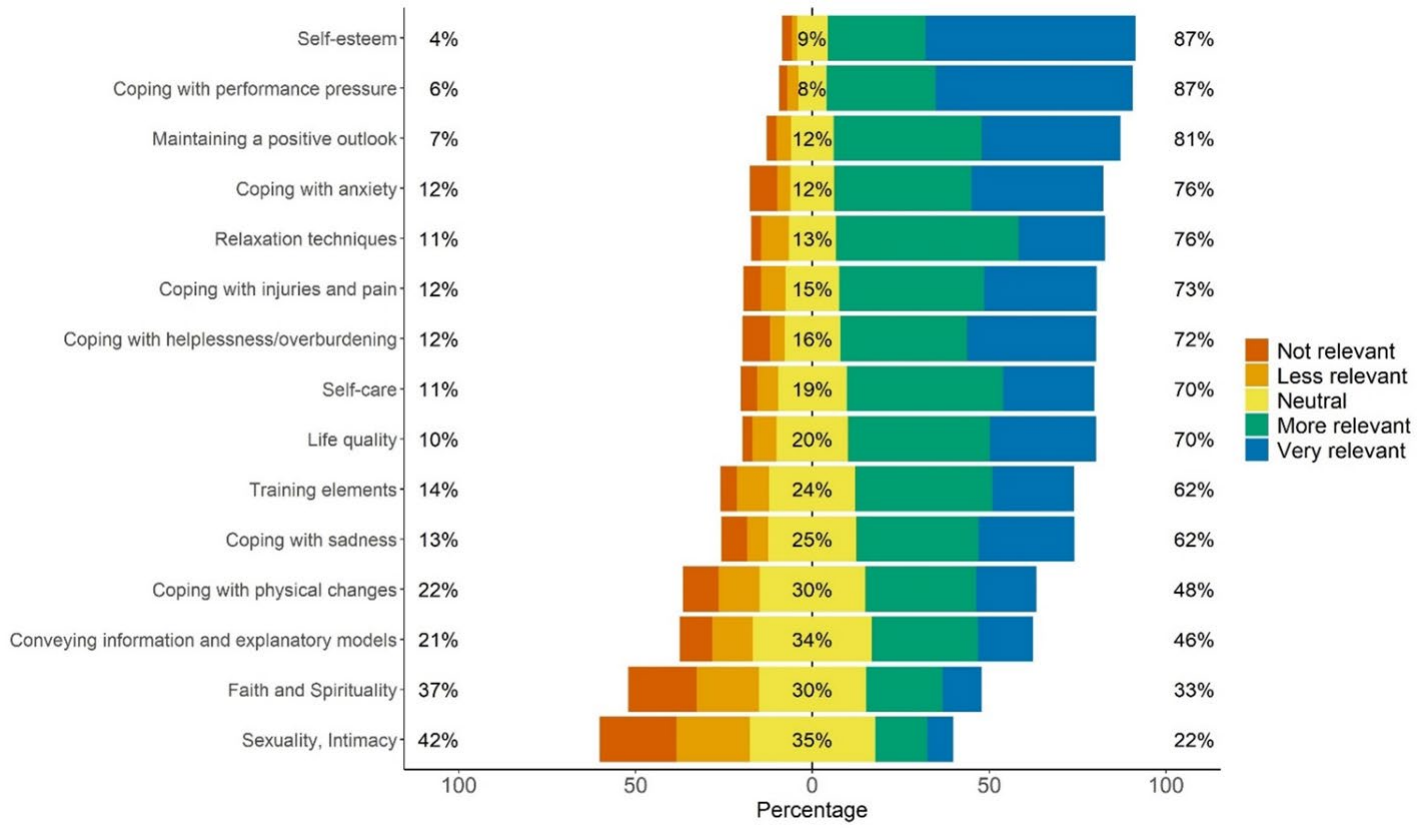
Distribution of participants’ ratings (in %) of content relevance for e-mental health interventions targeting adolescent elite athletes (*N* = 217)

A smartphone application was identified as the most favoured format, selected by 93.09% of the athletes, followed by audio/video materials (53.92%), and interactive tasks (52.07%). Regarding device preferences, 95.39% of the athletes indicated that the e-mental health intervention should be accessible via smartphone (see Table 5). The most relevant topics identified by adolescent elite athletes were self-esteem (87.14%), coping with performance pressure (86.64%), maintaining a positive outlook (81.11%), and managing anxiety (76.03%). A detailed overview of all responses on content relevance is presented in Fig. 1.

## Discussion

The present study investigated adolescent elite athletes’ acceptance, needs, and demands of e-mental health interventions, which may serve as a low-threshold support option for this vulnerable group. Notably, above 90.32% of the participants reported increased psychological distress, highlighting the need for targeted e-mental health interventions for this population. Gender and the UTAUT predictors, PE, EE, and SI significantly predicted acceptance in adolescent elite athletes. All predictors of the model explained 73.80% of acceptance, which is even higher compared to a previous study investigating acceptance in adult elite athletes [35]. More than half of the participants indicated high acceptance. Overall, acceptance of e-mental health interventions was moderate to high. E-mental health interventions for adolescent elite athletes that are tailored to the identified predictors may represent a promising support option in high demand.

Identifying predictors of acceptance provides valuable insights into the intention to use e-mental health interventions and has been examined across several cohorts [35, 38, 42, 44, 66, 67]. Compared to the high acceptance among adult elite athletes [35], adolescent athletes showed moderate to high overall acceptance. Although adolescents typically have strong digital skills [68], they might favour online technologies only in their function for social networking, entertainment, or gaming [69, 70] but not in improving their mental health. Adult elite athletes may have more awareness for mental health problems and thus view e-mental health interventions as practical and beneficial. In line with that, adult elite athletes are willing to use new technology when they recognize the practical benefits of e-mental health interventions [71], while adolescents elite athletes tend to be more sceptical and may perceive e-mental health interventions as less trustworthy . Viewing e-mental health interventions as ‘serious’ or ‘intended for adults’ may result in slightly lower acceptance among adolescent elite athletes than adult elite athletes [35]. This insight suggests that incorporating more age-specified elements, for example more video material or gamification elements, could enhance program appeal for younger users [41, 72].

In accordance with the results in the adult elite athletes’ sample, gender was a predictor for adolescent elite athletes’ acceptance [35]. Female athletes showed significantly higher acceptance towards e-mental health interventions than male athletes. This is in line with other studies, which address that, women utilize e-mental health interventions more frequently than men and exhibit fewer reservations about their use [70, 73, 74]. Female elite athletes also have previously been discussed to be more receptive for e-mental health interventions due to their higher psychological distress compared to their male counterparts [35]. This explanation is also supported by the result of the present study showing that the prevalence of psychological distress, anxiety, and depression symptoms are higher in female than in male adolescent elite athletes, which is also evident in other studies [52]. One could presume that individuals with higher levels of psychological burden could have a higher acceptance towards e-mental health interventions [35, 66].

This assumption is also underlined by the higher acceptance towards e-mental health interventions observed among adolescent elite athletes practising individual sports compared to those in team sports, consistent with findings in adult elite athletes [75]. Team sports are associated with lower rates of anxiety and depression [76], whereas adolescent elite athletes practicing individual sports may experience higher psychological strain, as reflected by the enhanced prevalence of anxiety or depressive symptoms reported in various studies [75]. This difference may stem from reduced social support, requiring individual sport athletes to cope with pressure largely on their own. In contrast, team sports can provide protective effects through supportive group dynamics, potentially reducing the need for additional mental health services [77]. In team sports, athletes’ willingness to use e-mental health interventions may be shaped by social dynamics and team norms [78], whereas individual athletes may feel less constrained by group decisions and thus more inclined to engage with such interventions [79]. However, the initial effect of sport type on acceptance was attenuated after accounting for UTAUT predictors, potentially supporting that differences in acceptance across sport types are mediated by underlying expectancy and social influence mechanisms rather than by sport type per se [39].

Furthermore, traditional gender roles and the fear of stigmatization may further reduce the acceptance of e-mental health interventions in male athletes and adolescent elite athletes practising team sports [80, 81]. Thus, the inherent anonymity offered by e-mental health interventions may act as convincing motivator for its use. Consequently, this feature should be carefully considered when developing, implementing, and disseminating e-mental health interventions to maximize uptake among male and team sport athletes.

Contrary to previous studies, psychological symptomatology did not predict acceptance [35, 66]. Considering that athletes of the next generation might use digital technologies as leisure activity instead of health improvement [41, 69, 72], anxiety, depression, and distress symptoms might not directly contribute to acceptance of e-mental health interventions. Short-form digital content such as videos, reels, and endless scrolling (‘doomscrolling’) can serve as a coping strategy to immediately reduce distress, for example by distracting users from their problems [82, 83]. In contrast, e-mental health interventions require active engagement with problems and psychological symptoms. This suggests that elite adolescent athletes may prefer easily accessible digital content with short-term stress-relieving effects over e-mental health interventions as a long-term strategy for stress management. The finding that internet anxiety, digital confidence, and digital overload did not predict acceptance might also suggest that adolescent elite athletes do not regard e-mental health interventions as a relevant or meaningful avenue for mental health support. A further elucidation could be the ceiling effect, due to the adolescent elite athletes’ strong digital skills [68, 84].

In line with the UTAUT the predictors PE, EE, and SI explained variance in acceptance of e-mental health interventions [39]. Consistent with previous studies, EE is a key predictor of acceptance in e-mental health interventions [57, 66]. Moreover, the provision of tutorials and support services may facilitate the initiation process and enhance the EE. In addition, e-mental health interventions offer the flexibility to be used between training sessions, without requiring the effort of in-person interventions, which in turn may increase their appeal to adolescent elite athletes. Finally, it is noteworthy that these predictors are interconnected, with EE functioning as a mediator that exerts a positive influence on PE, particularly in cases where EE is elevated [85].

SI plays a role regarding the acceptance, particularly when the social environment like the trainer or teammates support or endorse the use of technology [86]. Furthermore, the propensity to utilise e-mental health interventions could be influenced by the experiences of team colleagues or the counselling of the trainer [87]. This reinforces the importance of the trainer´s willingness to support e-mental health interventions. In addition, the utilisation of a given technology may be shaped by social pressures, especially when the trainer advocates for its implementation. Consequently, social dynamics may strengthen the adolescent elite athletes’ perception of e-mental health interventions as a helpful tool.

Other studies demonstrated that a negative PE is associated with a low acceptance rate [67], which is in line with our findings showing PE as the strongest predictor of acceptance. This highlights the importance of education about e-mental health interventions in adolescent elite sports. The utilisation of technologies that are tailored to the specific requirements of adolescent elite athletes could result in an increased perceived usefulness and, consequently, an enhanced perceived ease of use. Previous studies stated the importance of addressing these differences by tailoring programs to better meet the specific needs and preferences of each target group [41, 70].

Both adult and adolescent elite athletes showed similar preferences regarding the features and formats of e-mental health interventions, particularly favouring a smartphone-based application [88]. Offering 10 to 20-minute single sessions accessible via mobile phone or tablet may best meet adolescent elite athletes’ needs, allowing them to engage with the program flexibly during travel or between training sessions without imposing additional burden [29, 30]. This aligns with the finding that elite athletes across both age groups preferred a program that can be accessible on demand and adapted to their individual needs [88]. Furthermore, self-esteem and coping with aversive emotional experiences emerged as key topics of interest for both adult and adolescent athletes [88]. These findings suggest that the needs and demands across different elite athlete age groups are sufficiently consistent to support the use of comparable content and session durations in a smartphone-based intervention. Nevertheless, developers should account for potential differences related to gender and sport type (team versus individual) and ensure seamless integration of the e-mental health intervention into existing support systems, ideally facilitated by coaches or teammates.

This study offers valuable insights into adolescent elite athletes’ acceptance towards e-mental health interventions. However, several limitations should be considered. This cross-sectional study cannot provide any information on causalities. Moreover, the sample was not fully representative, with a higher proportion of female participants and unequal distribution across sports disciplines. Participation required internet access, possibly attracting athletes with higher media literacy or interest in digital tools. This potential selection bias could, however, also be seen as a strength, as it reflects the characteristics of the actual target group for e-mental health interventions. Data collection during the COVID-19 pandemic could have temporarily increased openness toward digital solutions [64, 89]. Another limitation is the reliance on self-reported data, which may be influenced by social desirability and question the participants’ eligibility [90, 91]. The anonymity of the online survey might have encouraged participants to respond honestly. For mental health assessment, validated and widely used instruments were employed (GAD-7, PHQ-8, and Distress Thermometer). These instruments are symptom-based and therefore limited in their diagnostic precision, but appropriate to screen mental strain and serve as predictors for acceptance in non-clinical populations such as adolescent elite athletes.

## Conclusions

This study emphasizes the need to incorporate adolescent elite athletes’ preferences into the design of e-mental health interventions to ensure effectiveness and sustained engagement. E-mental health interventions tailored to their specific needs can support mental health and coping strategies in this high-performance population. The moderate to high acceptance and articulated preferences identified in this study offer a solid basis for creating e-mental health interventions that are accessible in a flexible, low-threshold format and complement existing supportive structures. Important predictors of acceptance were gender and the UTAUT predictor’s performance expectancy, effort expectancy, and social influence. Preferred features enclosed short sessions available as a smartphone-based application. Effective implementation requires e-mental health interventions that are user-friendly and sensitive to stigma, emphasizing factors that promote acceptance rather than focusing solely on symptom severity. These results provide a foundation for future e-mental health interventions designed to fill critical gaps in mental healthcare for this vulnerable and high-risk population.

## Data Availability

The datasets generated during and/or analysed during the current study are available from the corresponding author on reasonable request.

## Abbreviations

UTAUT: Unified Theory of Acceptance and Use of Technology; etc
